# Supplementary material for: Essential Oil Composition Analysis of Cymbopogon Species from Eastern Nepal by GC-MS and Chiral GC-MS, and Antimicrobial Activity of Some Major Compounds
Source: Molecules. 2023 Jan 5;28(2):543. doi: 10.3390/molecules28020543 (PMC9863348; doi:10.3390/molecules28020543)
Supplement: Supplementary file 1 [file molecules-28-00543-s001.zip › molecules-2127943-supplementary.pdf]

Table S1: Chemical composition of *Cymbopogon winterianus* essential oils extracted from different parts

RI_{calc}	RI_{db}	Compounds	Root	Root hair & stalk	Leaves	Root stalk & shoot
850	848	(3*Z*)-Hexenol	-	-	t	-
865	867	4-Heptanone	-	-	t	-
921	921	Tricyclene	-	t	-	-
932	931	α-Pinene	-	t	-	t
946	948	Camphene	t	t	-	t
952	954	Benzaldehyde	t	t	-	-
969	967	Sabinene	-	t	-	-
981	982	6-Methyl-5-hepten-2-one	t	0.1	0.2	0.8
988	987	Myrcene	-	t	t	t
988	988	Dehydro-1,8-cineole	-	-	t	t
990	991	6-Methyl-5-hepten-2-ol	-	t	-	t
991	996	*trans*-Dehydroxy linalool oxide	-	-	-	t
998	1002	n-Octanal	-	t	-	0.1
1024	1027	Limonene	-	t	t	t
1025	1029	beta-Phellandrene	-	t	-	-
1028	1031	*o*-Menthan-8-ol	t	-	t	-
1030	1032	*p*-Menthan-8-ol	t	0.1	0.1	-
1032	1033	(*Z*)-β-Ocimene	-	t	0.2	0.3
1044	1044	(*E*)-β-Ocimene	-	t	0.2	0.2
1051	1051	Bergamal	t	t	0.1	-
1068	1069	4-Nonanone	t	0.4	0.6	0.4
1084	1084	Camphenilone	-	t	-	-
1086	1088	Terpinolene	-	t	-	-
1089	1089	4-Nonanol	t	t	t	-
1090	1092	Rosefuran	-	t	t	t
1095	1098	Linalool	0.1	0.5	0.5	0.8
1099	1104	α-Pinene oxide	-	-	0.1	t
1106	1106	*cis*-Rose oxide	-	t	t	-
1108	1108	α-Cyclocitral	-	-	-	t
1118	1122	Fenchol	-	-	t	-
1123	1123	*cis*-*p*-Menth-2-en-1-ol	t	t	-	-
1126	1126	β-Methyl cyclohexanethanol	t	t	t	-
1128	1127	*allo*-Ocimene	-	-	-	t
1137	1136	*epi*-Photocitral A	-	-	t	-
1136	1141	*trans*-*p*-Menth-2-en-1-ol	-	t	-	-
1140	1142	*exo*-Isocitral	-	t	t	0.1
1141	1144	Verbenol	-	t	-	-
1145	1145	Isopulegol	0.2	0.3	-	-
1145	1147	*p*-Menth-3-en-8-ol	t	-	-	-
1147	1148	*trans*-Chrysanthanal	-	-	0.3	-

1148	1151	Citronellal	1.1	8.6	13.7	0.3
1153	1154	*trans*-β-Terpineol	t	t	-	-
1155	1157	*iso*-Isopulegol	0.1	0.1	t	-
1160	1160	*cis*-Chrysanthenol	t	t	0.2	0.2
1160	1162	*iso*-borneol	t	t	-	-
1160	1164	*cis*-Dihydro-α-terpineol	-	t	-	-
1165	1166	*exo*-Acetoxy camphene	t	t	-	-
1167	1170	Borneol	0.3	0.7	-	t
1165	1168	Rosefuran epoxide	-	0.1	t	0.1
1174	1179	Terpinen-4-ol	t	t	-	-
1177	1178	*trans*-Isocitral	-	0.1	0.3	0.4
1179	1186	*p*-Cymen-8-ol	-	t	-	-
1193	1193	(4*Z*)-Decenal	-	-	t	-
1199	1195	α-Terpineol	0.2	0.5	t	t
1201	1206	Decanal	t	t	t	0.2
1207	1208	*trans*-Piperitol	-	t	-	-
1216	1216	*trans*-Isopiperitenol	-	-	-	t
1218	1219	Epoxy neral	-	t	t	t
1223	1222	Citronellol	t	t	0.2	t
1227	1223	Nerol	4.3	7.5	5.1	0.2
1232	1230	Epoxy geranial	-	t	0.1	0.1
1235	1238	Neral	1.2	6.5	8.3	31.5
1249	1250	Geraniol	0.1	8.6	37.1	0.4
1249	1253	Piperitone	-	-	-	t
1264	1268	Geranial	1.9	10.7	11.0	59.0
1270	1271	4-Undecanone	t	0.1	t	-
1271	1275	*trans*-Chrysanthenyl acetate	-	t	-	-
1273	1280	*trans*-Carvone oxide	-	0.1	t	0.2
1287	1282	Bornyl acetate	t	t	-	-
1290	1292	Dehydrobornyl acetate	-	t	-	-
1298	1296	Geranyl formate	-	t	t	-
1309	1308	*p*-Vinyl guaiacol	t	-	-	-
1309	1312	Methyl geranate	-	-	-	t
1311	1313	Carvenolide	t	0.1	0.1	-
1312	1316	Citronellic acid	0.1	0.1	t	-
1324	1327	Myrtenyl acetate	-	t	-	-
1335	1333	δ-Elemene	t	-	-	-
1337	1338	Citridiol A	-	t	-	-
1350	1347	Citronellyl acetate	t	1.9	1.9	-
1356	1350	Eugenol	0.7	1.2	1.0	-
1358	1353	Neric acid	t	-	-	0.4
1359	1356	Neryl acetate	-	t	t	-
1359	1358	9-Decenoic acid	t	-	-	-
1360	1360	(5*E*)-2,4,4,7-Tetramethylocta-5,7-dien-3-	-	-	-	0.1

		ol				
1373	1367	α-Ylangene	t	t	t	-
1375	1369	Decanoic acid	t	t	-	-
1374	1373	α-Copaene	t	-	-	-
1379	1376	Geranyl acetate	0.1	5.3	11.3	0.3
1382	1380	*cis*-β-Elemene	0.1	0.1	t	-
1387	1381	β-Bourbonene	-	-	t	-
1389	1387	*trans*-β-Elemene	2.1	0.9	0.3	t
1393	1393	Vanillin	-	t	-	-
1406	1401	(*Z*)-Isoeugenol	t	t	t	-
1408	1408	Dodecanal	t	t	-	t
1417	1417	(*E*)-β-Caryophyllene	0.3	0.6	2.0	0.2
1434	1427	γ-Elemene	t	t	t	-
1441	1432	β-Cedrane	t	-	-	-
1442	1438	6,9-Guaiadiene	t	t	-	-
1448	1441	*cis*-Muurola-3,5-diene	t	-	-	-
1448	1444	(*E*)-Isoeugenol	0.1	0.3	0.5	0.9
1452	1453	α-Humulene	t	0.1	0.3	t
1461	1459	*cis*-Cadina-1(6),4-diene	t	-	-	-
1465	1463	*cis*-Muurola-4(14),5-diene	-	t	t	-
1475	1471	Selina-4,11-diene	t	t	-	-
1475	1472	*trans*-Cadina-1(6),4-diene	t	t	-	-
1483	1479	α-Amorphene	-	t	-	-
1484	1480	Germacrene D	0.7	0.4	0.4	t
1484	1485	δ-Selinene	-	t	-	-
1489	1487	β-Selinene	0.2	0.1	-	-
1493	1490	*trans*-Muurola-4(14),5-diene	t	-	-	-
1498	1494	α-Selinene	0.1	-	-	-
1501	1496	α-Muurolene	t	t	-	-
1503	1501	β-Dihydroagarofuran	0.1	-	-	-
1506	1503	β-Cadinene	-	-	t	-
1508	1504	Germacrene A	t	t	-	-
1513	1509	γ-Cadinene	0.7	0.7	0.7	0.1
1514	1511	Cubebol	-	t	-	t
1516	1516	δ-Cadinene	0.1	-	t	t
1519	1519	Guaiacyl acetone	t	0.1	-	0.2
1528	1524	(*E*)-γ-Bisabolene	-	-	-	t
1531	1529	(*Z*)-Nerolidol	-	-	-	t
1533	1533	10-*epi*-Cubenol	-	-	0.1	-
1537	1535	α-Cadinene	t	t	t	-
1544	1539	α-Calacorene	t	-	-	-
1548	1548	α-Elemol	53.1	29.5	1.9	1.2
1553	1553	Geranyl butyrate	-	0.1	t	-
1558	1557	Germacrene B	-	t	t	-

1574	1574	Germacrene D-4-ol	-	0.1	-	-
1582	1577	Caryophyllene oxide	0.1	0.2	0.3	t
1592	1592	Viridiflorol	-	0.3	t	t
1600	1594	Guaiol	t	-	-	-
1607	1604	5-*epi*-7-*epi*-α-Eudesmol	0.3	0.4	-	-
1608	1608	Humulene epoxide II	-	-	t	-
1610	1611	Sesquilavandulol	0.4	0.2	t	-
1618	1613	1,10-di-*epi*-Cubenol	t	0.1	t	t
1621	1621	10-*epi*-γ-Eudesmol	0.6	0.3	-	-
1627	1626	1-*epi*-Cubenol	-	0.1	-	-
1629	1628	Eremoligenol	0.6	0.3	-	-
1630	1630	γ-Eudesmol	7.5	2.0	0.1	t
1640	1638	Hinesol	0.3	0.2	-	-
1642	1640	α-Muurolol	-	0.1	-	-
1643	1642	*epi*-α-Muurolol	-	0.1	-	-
1647	1644	δ-Cadinol	-	0.1	-	-
1649	1647	β-Eudesmol	0.1	0.1	0.2	t
1652	1659	α-Eudesmol	18.9	5.3	-	-
1658	1660	Selin-11-en-4α-ol	0.4	0.6	-	-
1662	1661	7-*epi*-α-Eudesmol	0.1	0.1	-	-
1666	1663	14-Hydroy-*epi*-(*Z*)-caryophyllene	-	-	-	0.1
1667	1667	Intermedeol	-	0.1	-	-
1670	1672	Bulnesol	0.1	t	-	-
1672	1673	*iso*-Bulnesol	t	-	-	-
1678	1679	*iso*-Hedycariol	t	-	-	-
1688	1683	2,3-Dihydro farnesol	0.1	0.1	-	-
1700	1696	Eudesm-7(11)-en-4-ol	t	-	-	-
1713	1706	(2*E*,6*Z*)-Farnesal	0.2	0.1	t	-
1714	1714	Pentadecanal	t	-	-	-
1740	1734	(2*E*,6*E*)-Farnesal	0.3	0.2	t	-
1792	1789	8α-Acetoxyelemol	t	0.1	-	-
1798	1793	*epi*-Cryptomeridiol	0.1	t	-	-
1813	1814	Cryptomeridiol	0.2	0.1	-	-
1832	1832	Neophytadiene	-	-	0.1	-
2001	1997	Citriodiol acetal	t	-	-	-
2683	2693	Dehydrodisoeugenol	t	0.3	-	0.2

RI_{calc}: retention index determined with respect to a homologous series of n-alkanes on a ZB-5 ms column and RI_{db}: retention indices from the databases [1,2]. “t” indicates trace (≤0.05%) and “-” indicates not detected.

Table S2: Chemical composition of *Cymbopogon citratus* essential oil extracted from roots and leaves

			Lemongrass	
RI_{db}	RI_{calc}	compounds	Root	Leaves
982	981	6-Methyl-5-hepten-2-one	0.4	0.2
991	989	6-Methyl-5-hepten-2-ol	t	-
1002	998	n-Octanal	-	t
1027	1024	Limonene	-	t
1031	1026	1,8-Cineole	-	0.1
1033	1032	(*Z*)-β-Ocimene	t	t
1044	1044	(*E*)-β-Ocimene	t	t
1051	1051	Bergamal	-	t
1069	1068	4-Nonanone	0.1	0.1
1092	1090	Rosefuran	t	0.1
1098	1095	Linalool	0.5	0.6
1120	1119	*trans*-*p*-Mentha-2,8-dien-1-ol	t	-
1135	1136	*cis*-*p*-Mentha-2,8-dien-1-ol	t	-
1136	1137	*epi*-Photocitral A	-	0.1
1142	1140	exo-Isocitral	t	0.1
1145	1145	Isopulegol	t	-
1148	1147	*trans*-Chrysanthanal	-	0.2
1151	1148	Citronellal	0.1	0.1
1160	1160	*cis*-Chrysanthenol	0.1	0.2
1168	1165	Rosefuran epoxide	t	0.2
1170	1166	*p*-Mentha-1,5-dien-8-ol	0.1	-
1178	1177	*trans*-Isocitral	0.4	0.4
1181	1180	Anethofuran	0.1	-
1195	1199	α-Terpineol	t	-
1205	1200	β-Cyclocitral	0.1	-
1206	1201	n-Decanal	-	0.2
1216	1216	*trans*-Isopiperitenol	t	-
1219	1218	Epoxy neral	t	0.1
1222	1223	Citronellol	0.3	-
1223	1227	Nerol	0.1	t
1230	1232	Epoxy geranial	0.1	0.1
1238	1235	Neral	16.6	36.1
1250	1249	Geraniol	0.4	0.3
1253	1250	Piperitone	-	t
1268	1264	Geranial	25.0	53.1
1280	1273	*trans*-Carvone oxide	t	0.4
1308	1309	*p*-Vinyl guaiacol	t	-
1312	1309	Methyl geranate	0.1	0.1
1353	1358	Neric acid	0.3	0.6
1360	1360	(5*E*)-2,4,4,7-Tetramethyl octa-5,7-dien-3-ol	-	0.1

1367	1373	α-Ylangene	-	t
1376	1379	Geranyl acetate	t	t
1379	1382	*cis*-β-Elemene	t	-
1387	1389	*trans*-β-Elemene	1.4	0.1
1408	1408	Dodecanal	-	t
1417	1417	(*E*)-β-Caryophyllene	0.2	1.0
1444	1448	(*E*)-Isoeugenol	0.6	0.4
1453	1452	α-Humulene	t	0.1
1474	1475	Selina-4,11-diene	t	-
1476	1475	(*E*)-beta-Ionone	-	t
1478	1475	γ-Gurjunene	t	-
1480	1484	Germacrene D	-	0.1
1487	1489	β-Selinene	0.1	-
1504	1504	Cuparene	0.1	-
1509	1513	γ-Cadinene	0.3	0.8
1511	1514	Cubebol	-	t
1516	1516	δ-Cadinene	-	t
1519	1519	Guaiacyl acetone	t	0.1
1524	1528	(*E*)-γ-Bisabolene	0.2	0.1
1532	1532	γ-Cuprenene	t	-
1533	1533	10-*epi*-Cubenol	-	0.1
1548	1548	α-Elemol	31.5	0.3
1577	1582	Caryophyllene oxide	t	1.3
1592	1592	Viridiflorol	-	t
1604	1607	5-*epi*-7-*epi*-α-Eudesmol	0.1	-
1608	1608	Humulene epoxide II	-	0.1
1613	1618	1,10-di-*epi*-Cubenol	-	t
1621	1621	10-*epi*-γ-Eudesmol	0.3	-
1630	1630	γ-Eudesmol	5.2	t
1638	1640	Hinesol	0.2	-
1641	1641	Valerianol	t	-
1642	1644	*epi*-α-Muurolol	-	t
1647	1649	β-Eudesmol	0.1	0.1
1659	1652	α-Eudesmol	11.3	-
1661	1662	7-*epi*-α-Eudesmol	0.1	-
1663	1666	14-Hydroxy-*epi*-(*Z*)-caryophyllene	1.0	0.1
1668	1669	Isospathulenol	0.1	-
1683	1683	α-Bisabolol	0.1	-
1706	1713	(2*E*,6*Z*)-Farnesal	0.3	0.1
1713	1714	(2*E*,6*Z*)-Farnesol	-	-
1734	1740	(2*E*,6*E*)-Farnesal	0.5	t
1771	1770	8-α-Acetoxy elemol	0.1	-
1775	1773	*epi*-Cryptomeridiol	0.1	-
1814	1814	Cryptomeridiol	0.3	-

1832	1832	Neophytadiene	-	0.5
2693	2683	Dehydrodisoeugenol	0.2	-

RI_{calc}: retention index determined with respect to a homologous series of n-alkanes on a ZB-5 ms column and RI_{db}: retention indices from the databases [1,2]. "t" indicates trace (≤0.05%) and "-" indicates not detected.

Table S3: Chemical composition of *Cymbopogon martini* essential oils extracted from roots and leaves

			Palmarosa	
RI_{db}	RI_{calc}	Compounds	Root	Leaf
759	762	Pentanol	t	t
848	850	(3Z)-Hexenol	-	0.1
859	854	(2E)-Hexenol	-	t
862	863	n-Hexanol	-	t
871	869	Isopentyl acetate	-	t
901	899	3-Methyl cyclopentyl acetate	-	t
982	981	6-Methyl-5-hepten-2-one	-	t
987	988	Myrcene	0.1	0.2
1027	1024	Limonene	t	t
1031	1026	1,8-Cineole	-	t
1033	1032	(Z)-β-Ocimene	t	0.2
1044	1044	(E)-β-Ocimene	0.2	0.9
1049	1048	2,6-Dimethyl-2,6-octadiene	-	t
1051	1051	Bergamal	-	t
1059	1059	Acetophenone	t	-
1068	1067	cis-Linalool oxide (furanoid)	-	t
1070	1069	n-Octanol	t	t
1083	1085	p-Mentha-2,4(8)-diene	-	t
1087	1086	trans-Linalool oxide (furanoid)	-	t
1092	1090	Rosefuran	-	t
1093	1092	α-Pinene epoxide	-	t
1098	1095	Linalool	3.2	2.0
1127	1128	allo-Ocimene	-	t
1164	1167	Octanoic acid	t	-
1195	1199	α-Terpineol	t	t
1219	1218	Epoxy neral	-	t
1223	1227	Nerol	t	0.1
1230	1232	Epoxy geranial	-	t
1238	1235	Neral	t	0.2
1250	1249	Geraniol	87.9	76.6
1268	1264	Geranial	0.2	0.6
1296	1298	Geranyl formate	t	t
1331	1330	2,3-Dihydro-2,3-epoxy-Geraniol	t	t
1353	1358	Neric acid	t	-

1376	1379	Geranyl acetate	4.4	15.2
1387	1389	trans-β-Elemene	t	t
1417	1417	(E)-β-Caryophyllene	0.4	0.5
1445	1444	Geranyl acetone	t	-
1450	1451	(E)-β-Farnesene	t	t
1453	1452	α-Humulene	t	t
1468	1474	Geranyl propionate	-	t
1474	1475	Selina-4,11-diene	t	-
1476	1487	(E)-beta-Ionone	-	t
1487	1489	β-Selinene	t	t
1493	1496	Valencene	-	t
1502	1505	(E,E)-α-Farnesene	t	t
1520	1520	7-epi-α-Selinene	t	t
1548	1548	α-Elemol	t	t
1553	1557	Geranyl butanoate	t	0.2
1559	1561	(E)-Nerolidol	0.1	0.1
1577	1582	Caryophyllene oxide	t	0.1
1596	1606	Geranyl isovalerate	t	t
1611	1611	Tetradecanal	t	-
1652	1648	Ageratochromene	t	-
1653	1651	Pogostol	-	t
1660	1658	Selin-11-en-4-α-ol	-	t
1671	1671	n-Tetradecanol	t	-
1713	1714	(2E,6Z)-Farnesol	3.0	1.3
1734	1740	(2E,6E)-Farnesal	t	t
1749	1752	Geranyl heptanoate	0.1	1.0
1830	1821	Farnesyl acetate	t	0.1
1832	1832	Neophytadiene	-	t
1839	1838	Phytone	t	-
1941	1943	Geranyl octanoate	t	0.2
2698	2700	Heptacosane	t	-

RI_{calc}: retention index determined with respect to a homologous series of n-alkanes on a ZB-5 ms column and RI_{db}: retention indices from the databases [1,2]. “t” indicates trace (≤0.05%) and “-” indicates not detected.

References

1. Satyal, P. Development of GC-MS Database of Essential Oil Components by the Analysis of Natural Essential Oils and Synthetic Compounds and Discovery of Biologically Active Novel Chemotypes in Essential Oils, 2015.
2. Adams, R.P. *Identification of Essential Oil Components by Gas Chromatography/Mass Spectrometry*; 4th ed.; Allured Publishing: Carol Stream, IL, USA, 2007.
